# Costing of National STI Program Implementation for the Global STI Control Strategy for the Health Sector, 2016-2021

**DOI:** 10.1371/journal.pone.0170773

**Published:** 2017-01-27

**Authors:** Eline L. Korenromp, Teodora Wi, Stephen Resch, John Stover, Nathalie Broutet

**Affiliations:** 1 Avenir Health, Geneva, Switzerland / Glastonbury, Connecticut, United States of America; 2 World Health Organization, Geneva, Switzerland; 3 Harvard University School of Public Health, Boston, Massachusetts, United States of America; University of Washington Department of Global Health, UNITED STATES

## Abstract

**Introduction:**

In 2016 the World Health Assembly adopted the global strategy on Sexually Transmitted Infections (STI) 2016–2021 aiming to reduce curable STIs by 90% by 2030. We costed scaling-up priority interventions to coverage targets.

**Methods:**

Strategy-targeted declines in *Chlamydia trachomatis*, *Neisseria gonorrhoeae*, *Treponema pallidum* and *Trichomonas vaginalis* were applied to WHO-estimated regional burdens at 2012. Syndromic case management was costed for these curable STIs, symptomatic Herpes Simplex Virus 2 (HSV-2), and non-STI vaginal syndromes, with incrementally expanding etiologic diagnosis. Service unit costs were multiplied with clinic attendances and people targeted for screening or prevention, by income tier. Human papilloma virus (HPV) vaccination and screening were costed for coverage increasing to 60% of 10-year-old girls for vaccination, and 60% of women 30–49 years for twice-lifetime screening (including clinical follow-up for positive screens), by 2021.

**Results:**

Strategy implementation will cost an estimated US$ 18.1 billion over 2016–2021 in 117 low- and middle-income countries. Cost drivers are HPV vaccination ($3.26 billion) and screening ($3.69 billion), adolescent chlamydia screening ($2.54 billion), and antenatal syphilis screening ($1.4 billion). Clinical management—of 18 million genital ulcers, 29–39 million urethral discharges and 42–53 million vaginal discharges annually—will cost $3.0 billion, including $818 million for service delivery and $1.4 billion for gonorrhea and chlamydia testing. Global costs increase from $2.6 billion to $ 4.0 billion over 2016–2021, driven by HPV services scale-up, despite vaccine price reduction. Sub-Saharan Africa, bearing 40% of curable STI burdens, covers 44% of global service needs and 30% of cost, the Western Pacific 15% of burden/need and 26% of cost, South-East Asia 20% of burden/need and 18% of cost.

**Conclusions:**

Costs of global STI control depend on price trends for HPV vaccines and chlamydia tests. Middle-income and especially low-income countries need increased investment, innovative financing, and synergizing with other health programs.

## Introduction

In 2016, the 69th World Health Assembly adopted a new global health sector strategy on Sexually Transmitted Infections (STI) for 2016–2021 [1]. The strategy aligns with the 2030 Agenda for Sustainable Development, and was based on achievements and lessons learned from the preceding global strategy covering 2006 to 2015 [2, 3]. The 2016–2021 strategy was developed by the World Health Organization (WHO) together with the global health sector strategies on HIV/AIDS and viral hepatitis, through a broad consultative process in 2015. Three organizing frameworks provided a common structure for the three strategies: universal health coverage, the continuum of health services and the public health approach. The STI strategy provides a framework for joint WHO and Member States actions at global, regional and country levels. The strategy outlines priority actions to ensure that the health sector response on STIs is scaled-up and strengthened so that progress towards ending the epidemic becomes a reality and to ensure that people-centered approaches help secure sustainable financing for relevant services, interventions and programmes in the future.

A concerted effort to rapidly scale up effective interventions and services will be needed to achieve the goal of ending STI epidemics as a major public health concern by 2030. The strategy therefore set as targets a 90% reduction in incidence rates of syphilis and gonorrhea from 2018 as the baseline, less than 50 cases of congenital syphilis per 100,000 live births in 80% of countries, and a sustained 90% national coverage and at least 80% in every district in countries which have HPV vaccination in their national immunization programmes [1].

In order to achieve these targets by 2030, the following milestones were set for 2020 to spur and measure progress:

- 70% of countries have STI surveillance systems in place able to monitor progress towards STI targets;
- 70% of countries have at least 95% of pregnant women screened for syphilis and HIV after informed consent, and 95% of pregnant syphilis-seropositive women are treated with at least one dose of intramuscular benzathine penicillin or other effective regimen;
- 70% of key populations have access to a full range of STI & HIV services, including condoms;
- 70% of countries provide STI services or links to such services in all primary, HIV, reproductive health, family planning and ante- and post-natal care services;
- 70% of countries deliver HPV vaccines through their national immunization programmes, and in those countries 90% national coverage in at least 80% of every district (or equivalent administrative unit); and
- 70% of countries report on antimicrobial resistance in *Neisseria gonorrhea*.

Implementation of the STI Strategy will generate important health impacts and returns on investment in health and country economies. HPV vaccination of girls and screening of women of reproductive age will generate considerable health care and productivity savings in future years by preventing cervical cancers. HPV vaccination is expected to reduce the lifetime risk of cervical cancer by 70%, because HPV types covered by the vaccine cause about 70% of cervical cancers [4]. Twice-lifetime HPV screening was conservatively estimated to reduce cervical cancers incidence by 20–25%. The resulting economic returns depend on the cost of cervical cancer treatment (around US$ 1,500–15,000 per patient) and on treatment coverage. Considering health gains and care savings, HPV vaccination and screening are estimated to be highly cost-effective in LMIC, at around US$300 per DALY averted [5, 6]. Benefits from improving STI control and reducing STI rates by 90% according to the Strategy’s target for 2030 will further include health care savings from STI episodes averted that incur economic productivity losses, morbidity and mortality due to infertility, pregnancy and congenital complications and psychosocial impacts.

This analysis estimates the costs of reaching the 2020 STI strategy milestones for the period 2016–2021, to support policy, planning, implementation, and future cost-benefit evaluation of the global STI strategy 2016–2021.

## Methods

Costs were estimated for priority actions and intervention based on the 2020 coverage targets and milestones across 117 low and middle-income countries (LMIC). Service requirements are based on the WHO’s estimates of regional burdens of four curable STIs (*Chlamydia trachomatis*, *Neisseria gonorrhoeae*, *syphilis* and *Trichomonas vaginalis)* [7] in 2012, extrapolated to future years according to the strategy’s expected burden reductions. Clinical management is costed for these four STIs, as well as *Herpes Simplex Virus* type 2 (HSV-2), bacterial vaginosis (BV) and *Mycoplasma genitalium* (Mycoplasma), with the syndromic approach as the recommended basis for case management throughout 2021. In addition, the costing includes vaccination and screening of Human Papilloma Virus (HPV), and screening for the curable STIs according to the 2020 coverage milestones.

STI prevention, essential health systems activities and related technical support are costed to the extent that they are not funded through programs for HIV/AIDS and hepatitis (which have been costed in the WHO’s corresponding 2016–2021 HIV/AIDS and hepatitis global strategies).

### STI case management

Numbers of STI cases and syndromes needing case management were estimated based on the WHO’s latest (2012) global and regional STI burden estimates [7]. For activities targeting STI patients or suspected patients, numbers of curable STI episodes (gonorrhea, syphilis, chlamydia and trichomoniasis) were assumed to be the same every year over 2015–2018 as in 2012, and to fall linearly by 23% between 2018 and 2021, consistent with the Strategy target of reducing the incidence of gonorrhea and syphilis by 90% from 2018 as the baseline to 2030. In contrast, numbers of STI clinic visits for symptoms due to HSV-2, BV and mycoplasma were assumed to be constant over 2016–2021, for two reasons. First, the strategy does not stipulate reductions in these conditions, which are less responsive to STI prevention, screening and treatment interventions [8, 9]. Second, any hypothetical reduction in the population-level burdens of these genitourinary conditions (which for BV and mycoplasma has not been estimated at global level) may be more than compensated if strategy-based improvements in treatment seeking and treatment access would increase the proportion of BV, mycoplasma and HSV-2-attributable ulcer episodes that would present at clinics (Table 1).

**Table 1:** Estimated annual STI syndrome numbers and case management volumes in 2015 and 2021.

*STI & syndrome episodes*: *volumes in need & treated*:	*2015*				*2021*				Comment & explanation, on:	
	*M*	*F*	*M*	*F*	*M*	*F*	*M*	*F*	2015 baseline	2021 Strategy target
**Syphilis: ASYMPTOMATIC**			**1,128,191**	**1,107,659**	***100%***	***100%***	**874,348**	**858,435**	Globally, 60% of syphilis episodes symptomatic (Newman 2015). In 2012, 927936 pregnant women had probable active syphilis; 779079 of them attended ANC; of those 300621 were tested & treated, 439412 not tested, and 39046 tested but not treated (Wijesooriya, 2016).	For 2021, assume 23% reduction from 2018 in syphilis numbers, including among ANC
Syphilis: Asymptomatic cases identified by ANC screening	0%	11%	-	120,248	NA	22%	NA	191,294		
Syphilis: Asymptomatic cases identified by MARPs screening	0%	0%	-	-	6.0%	4.9%	52,555	41,978	Prevalence in overall population incl. MARPs = 110% of prevalence in low-risk population (WHO global estimates, Newman 2015)	
Syphilis: Asymptomatic cases identified & treated, by clinic-based testing of GUD cases	1.1%	1.3%	12,438	14,324	6.8%	3.4%	59,380	29,404		
Syphilis: Asymptomatic cases identified & treated, by clinic-based testing of UD/VD cases					14%	19%	123,454	166,237		
Syphilis: Asymptomatic cases treated presumptively, during non-syphilis GUD, UD or VD	5.5%	6.5%	62,192	71,620	0%	0%	-	-		WHO 2016 Treatment guidelines: Screen GUD for syphilis
Syphilis: Asymptomatic cases NOT identified and/or treated	93.4%	81%	1,053,562	901,466	73%	50%	762,413	787,053		
**Syphilis: SYMPTOMATIC**	60%	60%	**1,692,287**	**1,661,488**	***100%***	***100%***	**1,311,523**	**1,287,653**	Globally, 60% of syphilis episodes symptomatic (Newman 2015).	
Syphilis: Symptomatic cases identified by ANC screening	0%	11%	-	180,373	NA	22%	NA	286,941	See above, under 'Asymptomatic cases identified by ANC screening'	
Syphilis: Symptomatic cases identified by MARPs screening	0%	0%	-	-	6%	5%	78,832	62,967		
Syphilis: Symptomatic cases presenting at an STI or PHC clinic, with GUD	55%	55%	930,758	913,818	66%	51%	862,883	656,422	WHO global estimates (Newman 2015)	
Syphilis: Symptomatic cases NOT identified and/or treated	45%	34%	761,529	567,297	28%	22%	369,807	281,324		
**Syphilis A- + SYMPTOMATIC**			**2,820,479**	**2,769,147**			**2,185,871**	**2,146,089**	WHO global estimates (Newman 2015)	
**Gonorrhea: ASYMPTOMATIC**	***100%***	***100%***	**16,979,870**	**23,989,634**	***100%***	***100%***	**13,159,399**	**18,591,966**	Globally, 64% of male and 34% of female gonorrhea episodes symptomatic (Newman 2015)	
Gonorrhea: Asymptomatic cases identified & treated by screening MARPs	0%	0%	-	-	0.5%	0.7%	67,570	129,869	Prevalence in overall population incl. MARPs = 110% of prevalence in low-risk population (Newman 2015)	
Gonorrhea: Asymptomatic cases treated after clinic-based test during non-gonorrheal UD/VD	0.4%	0.3%	63,000	81,000	0.3%	2.6%	36,530	487,153		
Gonorrhea: Asymptomatic cases treated presumptively during non-gonorrheal UD/VD	1.5%	2.4%	247,712	567,483	1.9%	1.0%	255,711	194,861		
Gonorrhea: Asymptomatic cases NOT identified and/or treated	98.2%	97.3%	16,669,158	23,341,151	97%	96%	12,799,587	17,780,083		
**Gonorrhea SYMPTOMATIC**	64%	34%	**30,186,435**	**12,358,296**	***100%***	***100%***	**23,394,487**	**9,577,680**	Globally, 64% of male and 34% of female gonorrhea episodes symptomatic (Newman 2015)	
Gonorrhea: Symptomatic cases identified by screening MARPs	0%	0%	-	-	0.5%	0.7%	120,125	66,902	Prevalence in overall population incl. MARPs = 110% of prevalence in low-risk population (Newman 2015)	
Gonorrhea: Symptomatic cases presenting at STI/PHC clinic, with UD or VD	40%	30%	12,074,574	3,707,489	69%	69%	16,256,016	6,637,473		
Gonorrhea: Symptomatic cases identified	40%	30%	12,074,574	3,707,489	70%	70%	16,376,141	6,704,376		
Gonorrhea: Symptomatic cases NOT identified and/or treated	60%	70%	18,111,861	8,650,807	30%	30%	7,018,346	2,873,304		
**Gonorrhea A- + SYMPTOMATIC**			**47,166,304**	**36,347,930**			**36,553,886**	**28,169,646**	WHO global estimates (Newman 2015)	
**Chlamydia: ASYMPTOMATIC**			**28,578,551**	**56,423,286**	***100%***	***100%***	**22,148,377**	**43,728,047**	Globally, 54% of male and 17% of female chlamydia episodes symptomatic (Newman 2015)	
Chlamydia: Asymptomatic cases identified & treated, by screening MARPs	0%	0%			0.9%	1.0%	199,815	457,297		
Chlamydia: Asymptomatic cases identified & treated, by STI clinic-based testing for non-chlamydial UD/VD	0.4%	0.4%	104,134	242,984	0.6%	5.3%	131,596	2,301,354		
Chlamydia: Asymptomatic cases treated but not identified through STI clinic-based presumptive treatment for non-chlamydial UD/VD	1.8%	6.0%	520,669	3,401,771	4.2%	2.1%	921,174	920,541		
Chlamydia: Asymptomatic cases identified & treated, by screening adolescents	0%	0%	-	-	1%	1%	193,538	543,216		
Chlamydia: Asymptomatic cases NOT identified and/or treated	98%	94%	27,953,748	52,778,531	93%	90%	20,702,254	39,505,638		
**Chlamydia: SYMPTOMATIC**	54%	17%	**33,548,734**	**11,556,577**	***100%***	***100%***	**26,000,269**	**8,956,347**	Globally, 54% of male and 17% of female chlamydia episodes symptomatic (Newman 2015)	
Chlamydia: Symptomatic cases identified by screening MARPs	0%	0%	-	-	0.9%	1.0%	234,565	93,663		
Chlamydia: Symptomatic cases identified & treated, by screening adolescents	0%	0%	-	-	0.9%	1.2%	227,197	111,261		
Chlamydia: Symptomatic cases presenting at an STI or PHC clinic, with UD or VD	40%	30%	13,419,493	3,466,973	68.2%	67.7%	17,738,426	6,064,518		
Chlamydia: Symptomatic cases identified	40%	30%	13,419,493	3,466,973	70%	70%	18,200,188	6,269,443	WHO global estimates (Newman 2015)	
Chlamydia: Symptomatic cases NOT identified and/or treated	60%	70%	20,129,240	8,089,604	30%	30%	7,800,081	2,686,904		
**Chlamydia A- + SYMPTOMATIC**			**62,127,285**	**67,979,862**			**48,148,646**	**52,684,393**	WHO global estimates (Newman 2015)	
**Trichomoniasis: ASYMPTOMATIC**			**69,547,794**	**45,114,684**	***100%***	***100%***	**53,899,541**	**34,963,880**	Globally, 6.4% of male and 34% of female trichomoniasis episodes symptomatic (Newman 2015)	
Trichomoniasis: Asymptomatic cases identified & treated by screening MARPs	0%	0%	0	0	0%	0%	-	-		
Trichomoniasis: Asymptomatic cases identified & treated, by STI clinic-based testing among non-trichomonal UD/VD patients	0.01%	0.50%	5,400	225,000	0.1%	4.0%	75,314	1,415,614		
Trichomoniasis: Asymptomatic cases treated but not identified through STI clinic-based presumptive treatment for non-trichomonal UD/VD	0.1%	7.0%	53,081	3,152,686	0.1%	9.4%	50,209	3,303,098		
Trichomoniasis: Asymptomatic cases NOT identified and/or treated	99.9%	93%	69,489,313	41,736,999	100%	87%	53,774,018	30,245,169		
**Trichomoniasis SYMPTOMATIC**	**6.7%**	**34%**	**4,994,322**	**23,240,898**	***100%***	***100%***	**3,870,599**	**18,011,696**	Globally, 6.4% of male and 34% of female trichomoniasis episodes symptomatic (Newman 2015)	
Trichomoniasis: Symptomatic cases identified by screening (high-risk groups, ANC)	0%	0%	-	-	0%	0%	-	-		
Trichomoniasis: Symptomatic cases presenting at an STI or PHC clinic, with UD or VD	40%	30%	1,997,729	6,972,269	70%	70%	2,709,420	12,608,187		
Trichomoniasis: Symptomatic cases identified	40%	30%	1,997,729	6,972,269	70%	70%	2,709,420	12,608,187	WHO global estimates (Newman 2015)	
Trichomoniasis: Symptomatic cases NOT identified and/or treated	60%	70%	2,996,593	16,268,629	30%	30%	1,161,180	5,403,509		
**Trichomoniasis A- + SYMPTOMATIC**			**74,542,116**	**68,355,582**			**57,770,140**	**52,975,576**	WHO global estimates (Newman 2015)	
HSV-2: Symptomatic GUD episodes (treated and not treated)							29,190,000	29,190,000	Authors' inference, combining the etiology back-estimation & HSV-2 ulcer estimate by Looker 2015: 40–80 million GUD episodes from herpes	
HSV-2: Symptomatic GUD episodes identified / presenting to clinics	28%	28%			28%	28%				
GUD presenting at STI clinics: due to syphilis	11%	11%	930,758	1,094,191	10%	7%	862,883	656,422		Strategy target to reduce new syphilis and gonorrhea cases 90% by 2030 and 23% by 2021, from 2018 base, applied to chlamydia. Clinical GUD cases due to syphilis decrease slightly more than 23%, because some cases are captured by MARPs screening.
GUD presenting at STI clinics: due to chancroid	1%	1%	84,614	99,472	0%	0%	-	-		
GUD presenting at STI clinics: due to HSV-2	88%	88%	7,446,064	8,753,528	90%	93%	7,446,064	8,753,528		The estimated 16.2 million (M+F) herpetic GUD cases imply that 28% of all 58 million herpetic GUD episodes present to clinics
**GUD presenting at STI clinics: total**			**8,461,436**	**9,947,190**			**8,308,947**	**9,409,949**		
UD or VD presenting at STI clinics: due to gonorrhea	41%	9%	12,074,574	3,707,489	42%	12%	16,256,016	6,637,473		
UD or VD presenting at STI clinics: due to chlamydia	46%	8%	13,419,493	3,466,973	46%	11%	17,738,426	6,064,518		
UD or VD presenting at STI clinics: due to trichomonas	7%	17%	1,997,729	6,972,269	7%	24%	2,709,420	12,608,187		
UD or VD presenting at STI clinics: due to mycoplasma & other	7%	17%	1,997,729	6,972,269	5%	13%	1,997,729	6,972,269		
VD presenting at STI clinics: due to BV & candidiasis	NA	50%	NA	20,916,808	NA	39%	NA	20,916,808		
**UD and/or VD presenting at STI clinics: total**	**100%**	**100%**	**29,489,525**	**42,035,809**	**100%**	**100%**	**38,701,590**	**53,199,256**		
GUD presenting at STI clinics: treated for HSV-2	70%	70%	5,923,005	6,963,033	70%	70%	5,816,263	6,586,964		Strategy milestone for 2020
GUD presenting at STI clinics: treated for syphilis	50%	50%	4,230,718	4,973,595	7%	5%	604,018	459,495		
GUD presenting at STI clinics: treated for chancroid	50%	50%	4,230,718	4,973,59	0%	0%	-	-		Chancroid will be nearly extinct, so no costing for chancroid testing or treatment
GUD presenting at STI clinics: tested for syphilis	10%	10%	846,144	994,719	70%	70%	5,816,263	6,586,964		Strategy milestone for 2020
UD or VD presenting at STI clinics: treated for gonorrhea	40%	50%	11,795,810	21,017,904	70%	20%	27,091,113	10,639,851	WHO 2016 treatment guidelines: Treat all UD for NG and CT (and so for mycoplasma); treat trichomonas only in recurring UD	Strategy milestone for 2020
UD or VD presenting at STI clinics: treated for chlamydia	40%	70%	11,795,810	29,425,066	70%	20%	27,091,113	10,639,851		For VD: Treat trichomonas & BV; + only those with risk factor also treated for NG & CT (and so mycoplasma); both by azithromycin.
UD or VD presenting at STI clinics: treated for trichomoniasis	10%	50%	2,948,952	21,017,904	10%	70%	3,870,159	37,239,480		Strategy: Syndromic management for UD, until adequate tests available; treat VD for trichomonas & BV; + only those with risk factor also (with azithromycin) for NG, CT and mycoplasma.
UD or VD presenting at STI clinics: treated for mycoplasma & other	40%	70%	11,795,810	29,425,066	70%	20%	27,091,113	10,639,851	Coverage as for chlamydia: same drug azithromycin	
VD presenting at STI clinics: treated for BV & candidiasis	NA	70%	NA	29,425,066	NA	70%		37,239,480		Strategy milestone for 2020
UD or VD presenting at STI clinics: screened for syphilis	10%	10%	2,948,952	4,203,581	70%	70%	27,091,113	37,239,480		WHO Guidelines: Screen NG/CT cases for syphilis
UD or VD presenting at STI clinics: tested by NAAT (for gonorrhea and chlamydia)	8%	5%	3,000,000	3,000,000	10%	50%	3,870,159	26,599,628	WHO 2004 treatment guidelines recommended testing VD for NG & CT; but not currently implemented in most settings	Strategy: Syndromic management for men with UD (until adequate tests become available) + selective etiology testing for monitoring purposes, e.g. every 2 years 20% of patients
UD or VD presenting at STI clinics: tested by wet-mode (for trichomonas)	1%	3%	300,000	1,500,000	15%	30%	5,805,239	15,959,777		Test recurrent UD for trichomonas; test VD for trichomonas per WHO guideline

Assumptions made to estimate volumes of STI syndromes presenting for treatment were:

- At 2015, 88% of genital ulcer disease (GUD) is due to HSV-2; 11% is due to syphilis and 1% in chancroid, in both men and women [10–14].
- There are 417 million people 15–49 years-old living with HSV-2 infection worldwide, who suffer 40–80 million episodes of genital ulcer disease due to recurrent HSV-2 annually in 2012 [15]. This number was quantified as an annual 58 million herpetic GUD episodes including those seeking and not seeking care. Assuming that 88% of GUD presenting in clinics are due to HSV-2, this estimate implied that 28% of herpetic GUD episodes present for clinical treatment, which we deemed plausible [10].
- From 2016 onwards, chancroid is (nearly) eradicated [11–14], and no diagnosis or treatment is costed.
- The annual number of clinical Urethral Discharge (UD) and Vaginal Discharge (VD) episodes due to *Mycoplasma* and other reproductive (non-STI) infections equals the number of clinical trichomonas episodes in 2015, and VD episodes due to BV are three times the number of clinical trichomonas episodes, so as to reproduce typical etiological patterns of VD [11, 16–19] and UD [11, 20–23]. Unlike for trichomonas the annual number of clinical episodes of Mycoplasma and BV was assumed constant throughout 2021.
- Each syndrome has one primary etiology estimated as explained above. In addition, co-infections are considered and costed, by assuming that STI patients presenting for clinical treatment have prevalences of curable STIs other than the one causing their STI syndrome of 3-fold the corresponding prevalence in the total adult population [7] (resulting in co-infection rates of 5–15% across the syndromes and STIs).
- Female Sex Workers (FSW) and Men-having-Sex-with-Men (MSM) have 10-fold higher prevalence than low-risk general populations for syphilis and gonorrhea, and 6-fold higher prevalence for chlamydia; which at WHO-estimated national STI prevalences [7], and UNAIDS-estimated FSW and MSM population sizes [24, 25] resulted in overall national prevalences that are, on average, 10% higher than prevalence in low-risk general population, consistent with WHO-estimated national versus low-risk prevalences [7].

Clinical case management was costed assuming that most countries continue to implement syndromic case management, i.e. treating cases based on syndromes and their likely etiologies, rather than etiologic diagnosis for every patient. Numbers of syndromes presenting for treatment were estimated based on WHO’s regional estimates of syphilis, gonorrhea, chlamydia and trichomoniasis burden, proportions of these that are symptomatic and presenting for treatment [7], and typical etiological distributions of GUD, UD and VD [10–12, 23, 26]. Calculations considered WHO guidelines, which recommend syndromic STI management [27–29], supported by roll-out of etiological testing where feasible to inform adequacy of national syndromic treatment guidelines:

- All GUD cases are presumptively treated for HSV-2;
- All GUD, VD and UD cases are screened for syphilis and treated for syphilis if positive;
- All VD cases are treated for trichomoniasis and BV; those with a risk factor or with signs of cervical infection (judged by the clinician) are in addition treated for gonorrhea, chlamydia and mycoplasma;
- All UD cases are treated for gonorrhea and chlamydia;
- Recurrent UD (15% of all UD cases [30–32]) is tested for trichomoniasis, and treated for trichomonas if trichomonas-positive (assumed in 67% of recurrent UD [33–35], i.e. 10% of all UD cases);
- Etiological testing of UD cases for gonorrhea and chlamydia for monitoring purposes is implemented in STI clinics for an average 10% of patients every year.

We assumed each of these recommendations to be implemented for 70% of patients across LMIC from 2016 onwards (Table 1). This implied some strategic improvements from the 2015 situation (Table 1), for which time we estimated that as much as 50% of GUD was presumptively treated for both syphilis and chancroid, but only 10% screened for syphilis. Over 2016–2021, GUD was assumed to be treated for syphilis in 8% of male and 5% of female patients, based on a targeted 70% testing coverage and an estimated 11% and 7% of male and female GUD due to syphilis over 2016–2021, respectively [10–12]. The modelled proportion of GUD due to syphilis was reduced relative to 2015 for women but not for men, reflecting the strategy’s target to roll-out ANC-based syphilis screening and treatment to reach at least 95% of pregnant women in 70% of countries [1].

### Syphilis screening in antenatal care

We assumed there will be 107 million ANC clients in 2016 [36], increasing to 118 million by 2021, based on country reports through the Global AIDS Response Progress Reporting (GAPRR) system over 2013–2014 [26, 37] and 2% annual population growth [26, 37,38]. Across LMIC, there would be 719,150 pregnant women with probable active syphilis in 2021, 23% less than in 2012 [36]. According to the STI Strategy target, of these women 95% in 70% of the countries (so 66.5% of all pregnant women with active syphilis) would be screened and treated during ANC.

### STI screening and prevention in adolescents

Prevention services for adolescents were costed for 70% of 15–19 year-olds in LMIC, at US$ 0.2 per adolescent-year. An annual 5% of sexually active (i.e. higher-risk) adolescents were assumed to be screened for chlamydia, at US$ 10 per test (assuming service delivery costs to be borne by HIV/AIDS programs, and test unit cost halved from 2015 costs). Of those positive (assuming prevalence as in the general 15-49-years population [7]) 70% were treated, at US$ 3 per treatment including service delivery. We estimated numbers of sexually active adolescents by applying proportions of adolescents who reported to be sexually active from Demographic and Health Surveys [39, 40], to national population sizes [41].

### STI screening and prevention in high-risk groups

Prevention targeting high-risk groups was costed for 70% of FSW and MSM, at US$ 0.1 per person-year—cheaper than for adolescents, assuming that outreach costs are borne by HIV/AIDS programs. Population sizes were taken from recent UNAIDS national HIV/AIDS estimations [42, 43], totaling 4.9 million MSM and 4.0 million FSW in 2014–15 across LMIC.

Seventy percent of FSW and MSM are targeted for annual syphilis, gonorrhea and chlamydia screening, and of those found positive 70% are treated. Costs considered the same diagnostic and treatment commodities used in clinical STI management (Table 2), excluding delivery cost which we assumed to be borne by HIV/AIDS programs. Only for syphilis screening, the unit cost per FSW or MSM was slightly higher than for clinic-based case management (US$ 0.12 instead of US$ 0.10) to reflect the additional Rapid Plasma Reagin (RPR) test for cases positive on rapid diagnostic test. FSW and MSM are assumed to have prevalence 10-fold higher than national adult populations for syphilis and gonorrhea, and 6-fold higher for chlamydia (matching the WHO’s 2015 estimate that these groups add an average 10% prevalent cases to those in general low-risk populations [7]).

**Table 2:** Unit costs of diagnostic and treatment commodities (unit cost assumed equal across all countries).

STI or syndrome	Treatment	Dose per day	Treatment duration (days)	Drugs, per dose	Drugs per treatment	25% procu-rement	Service delivery	Drugs + service delivery
Herpes	Acyclovir 400 mg	3	7	$ 0.04	$ 0.84	$ 0.21	$ 10.00	**$ 11.05**
Syphilis	Benzathine PCN 2.4 M	1	3	$ 0.44	$ 1.32	$ 0.33	$ 10.00	**$ 11.65**
Gonorrhea	Ceftriaxone 250 mg	1	1	$ 0.57	$ 0.57	$ 0.14	$ 10.00	**$ 10.71**
Chlamydia & mycoplasma	Azithromycine 500 mg	2	1	$ 0.38	$ 0.76	$ 0.19	$ 10.00	**$ 10.95**
Trichomoniasis	Metronidazole 500 mg	4	1	$ 0.01	$ 0.04	$ 0.01	$ 10.00	**$ 10.05**
Candidiasis	Clotrimazole suppository 500 mg	1	1	$ 0.19	$ 0.19	$ 0.05		
Vaginal discharge	Metronidazole 500 mg+ Clotrimazole suppository 500 mg	4 1	1 1	$ 0.01 $ 0.19	$ 0.23	$ 0.06	$ 10.00	**$ 10.29**
	**Diagnostic test**							
Syphilis	RPR (Point of Care)				$ 0.10	Not used in isolation		
Syphilis	Rapid Diagnostic Test: for ANC & STI clinic patients screening (Point of Care)				$ 0.40	NA	$ 3.00	**$ 3.40**
Syphilis	Rapid Diagnostic Test with RPR confirmation: for screening FSW & MSM (Point of Care)				$ 0.50	NA	$ 3.00	**$ 3.50**
Gonorrhea & Chlamydia	NAAT: assuming a price reduction starting 2016, from US$ 20 as of 2016 (specimen collection at primary level; testing in secondary and tertiary level of care facilities)				$ 10.00	NA	$ 3.00	**$ 13.00**
Trichomoniasis	Wet mount (Point of Care)				$ 1.00	NA	$ 3.00	**$ 4.00**

Data sources: Unit costs for STI diagnostics and drugs were obtained from UNICEF, using the laboratory test or medicines recommended in WHO STI Treatment 2003 guidelines [27] with consideration on anticipated changes in the forthcoming 2016 STI treatment guidelines [28, 29].

### HPV vaccination and screening

HPV vaccination and screening were costed for coverage target levels explored in a recent modelling exercise [6]. For both vaccination and screening, coverage would increase from 10% to 60% of populations in need over 2016–2021, in a 10-year roll-out scenario across 102 LMIC with national populations above 1 million.

For vaccination the total need is 49 million 10-year old girls in 2016, increasing to 52 million by 2021; for screening, 750 million women 30–49 years increasing to 828 million women over 2016–2021, of whom 10% would require screening in any year, to achieve twice-lifetime screening. For vaccination this scenario is comparable with the STI Strategy target to cover 80% of adolescents in 80% of countries by 2030, and the milestone that 70% of countries deliver HPV vaccination through their national immunization program by 2020. Of all girls who turn 10 during 2016–2021, 35% would be vaccinated.

Vaccination unit costs was estimated at an average US$ 30, considering an optimistic (2-tiered) vaccine price scenario [6, 45] and including country-specific cost of primary health care visits estimated by the WHO’s cost effectiveness and strategic planning (CHOICE) department [47].

Early detection, by screening for women in target groups, followed by treatment of detected pre-cancerous lesions can prevent the majority of cervical cancers [48]. Cervical cancer screening was modelled to occur twice per life-time (the minimum frequency based on WHO guidelines [49, 50]). Costing included visual inspection with acetic acid at age 30 and 40 years for LMIC and HPV test or Papanicolaou (PAP) test for those upper-middle income countries where PAP test is already established. Patients positive on screening were treated with cryotherapy for most cases and loop electrosurgical excision procedure in a small fraction of cases. In the few settings using PAP, the cost of follow up colposcopy/biopsy was included. The average cost per woman screened (including treatment costs when applicable) was US$ 20 [6].

### In-country support activities

STI surveillance, monitoring and evaluation was costed assuming a prevalence survey and periodic assessment of STI syndrome etiologies at US$ 250,000 for 70% of LMIC each once over 2016–2021, and an annual US$ 15,000 cost for routine STI case reporting for 70% of LMIC.

Monitoring of gonococcal antimicrobial resistance was costed based on the 2016–2020 programme budget of the Gonococcal Antimicrobial Surveillance Programme by WHO-RHR, as of July 2015 [51].

### Service costing

Service unit costs were multiplied with volumes of STI episodes and people targeted for screening or prevention, stratified between income tiers (Table 3). For activities targeting the general population (such as prevention services and screening), a 2% annual population growth was assumed across all countries.

**Table 3:** Unit costs of STI-related service deliveries, with unit cost varying by income tier.

Income level	Low & middle (average)	Low-income	Lower-middle	Upper-middle	Source
Clinical STI treatment, service delivery	**$ 10.00**	$ 3.00	$ 8.00	$ 20.00	Authors’ estimate, based on cost gradients in UNAIDS Fast Track [44]
Clinical STI diagnosis, service delivery	**$ 3.00**	$ 0.90	$ 2.40	$ 6.00	Authors’ estimate, based on cost gradients in UNAIDS Fast Track [44]
Prevention services for adolescents	**$ 0.20**	$ 0.06	$ 0.16	$ 0.40	UNAIDS Fast Track estimates, for Youth prevention interventions [44]
ANC syphilis screening	**$ 0.40**	$ 0.12	$ 0.32	$ 0.80	UNAIDS Fast Track estimates, for STI treatment interventions [44]
ANC syphilis treatment	**$ 11.65**	$ 3.50	$ 9.32	$ 23.30	Authors’ estimate, based on cost gradients in UNAIDS Fast Track [44]
Syphilis treatment for FSW, MSM or Transgender diagnosed by screening	**$ 22.20**	$ 3.50	$ 9.30	$ 23.30	Authors’ estimate, based on cost gradients in UNAIDS Fast Track [44]
NG treatment for FSW, MSM or Transgender diagnosed by screening	**$ 22.20**	$ 3.20	$ 8.60	$ 21.40	Authors’ estimate, based on cost gradients in UNAIDS Fast Track [44]
CT treatment for FSW, MSM or Transgender diagnosed by screening	**$ 22.20**	$ 3.30	$ 8.80	$ 21.90	Authors’ estimate, based on cost gradients in UNAIDS Fast Track [44]
CT treatment for adolescents diagnosed by screening	**$ 22.20**	$ 3.30	$ 8.80	$ 21.90	Authors’ estimate, based on cost gradients in UNAIDS Fast Track [44]
HPV vaccination	**$ 22.20**	$ 9.06	$ 24.15	$ 60.38	[6, 45]
HPV screening & preventive treatment	**$ 22.20**	$ 4.10	$ 10.20	$ 36.10	[6, 46]

Unit costs for STI diagnostics and drugs were obtained from UNICEF, using the laboratory test or medicines recommended in WHO STI Treatment 2003 guidelines [27] with consideration on anticipated changes in the forthcoming 2016 STI treatment guidelines (Table 2)[28, 29]. Where there were two or more WHO-recommended medicines, the more expensive one was selected. For medicines, a 25% overhead was added to account for procurement and transport.

Service delivery was costed at US$ 10 per treatment course, and US$ 3 per diagnosis. This is in line with a meta-analysis of clinical STI treatment cost in LMIC, which found a median of US$ 17.8 for treatment + drugs, in 2004 US$, across studies conducted in the 1990s and early 2000s [52] and slightly below assumptions in the UNAIDS 2014 Fast Track costing for STI treatment (US$ 7–53 across 6 WHO regions, and US$ 5–59 across LMIC with median US$ 24, per episode diagnosed and treated including plus commodity costs [43, 53,54, 55].

For ANC syphilis screening, we assumed a unit cost of $1.4 covering the test cost, with service delivery cost imbedded within maternal, neonatal and child health care (MNCH). Syphilis treatment following screening during ANC was costed at US$ 3.7 per treatment (considering only the medicine cost, with service delivery assumed under MNCH), in line with a WHO-commissioned cost-effectiveness and investment case analysis of syphilis screening and treatment in ANC [54, 55].

Across country income categories, delivery of clinical STI diagnosis and treatment was costed using a gradient in service unit costs, assuming 0.3-fold of the global weighted average cost for low-income countries, 0.8-fold global cost for lower-middle income countries and 2-fold global cost for upper-middle income countries (Table 3). This is in line with cost gradients by income group used in 2014 by UNAIDS for its global Fast Track costing to end the AIDS epidemic [43, 53] and by the WHO CHOICE [47]. The same gradient across income groups was applied for prevention services for adolescents, syphilis screening and treatment in ANC, STI screening and treatment in high-risk groups, and for chlamydia treatment for adolescents diagnosed by screening. For HPV vaccination and screening, the cost gradient was taken from the HPV control scenario modelling study that also provided the scale-up targets [6]. All other activities were assumed to have a fixed unit cost across all countries. Within a country, unit costs were assumed to be fixed every year over 2016–2021.

An overhead cost of 14% was applied to all country implementation costs, representing program enablers (planning and coordination, administration, supplies and logistics, staff training, monitoring and evaluation including surveillance and information systems) that were not costed explicitly in either the STI or HIV global 2016–2021 strategy. This percentage is the amount estimated to be spent on these functions by HIV/AIDS programs, and that was assumed in the 2014 UNAIDS Fast Track framework [43, 56].

### Allocation of global targets and costs to countries

Since the global STI strategy specified most of the targeted interventions and STI burden reductions only as global aggregates (i.e. mostly as 70% of countries, without specification of which countries), and it specified several intervention targets without direct link to which STIs this would impact (e.g. prevention services for adolescents or for FSW and MSM, surveillance), costing was first done at a global level, and then allocated to regions and countries according to their share in the global STI burden. First we attributed to each region its share in the overall global STI burden, based on WHO-estimated prevalence of the four curable STIs [7] and these STIs’ respective shares in global DALY burden according to the Global Burden of Disease 2013, with an average 5.5 DALYs per prevalent case of chlamydia, 11 DALYs for gonorrhea, 1.1 DALYs for trichomonas and 640 DALYs for syphilis (reflecting tertiary syphilis in adults, not congenital syphilis) [57]. Within regions, STI burdens then were allocated across countries based on each country’s share in the regional population 15–49 years.

## Results

### STI syndromes presenting for clinical treatment

**Fig 1:**
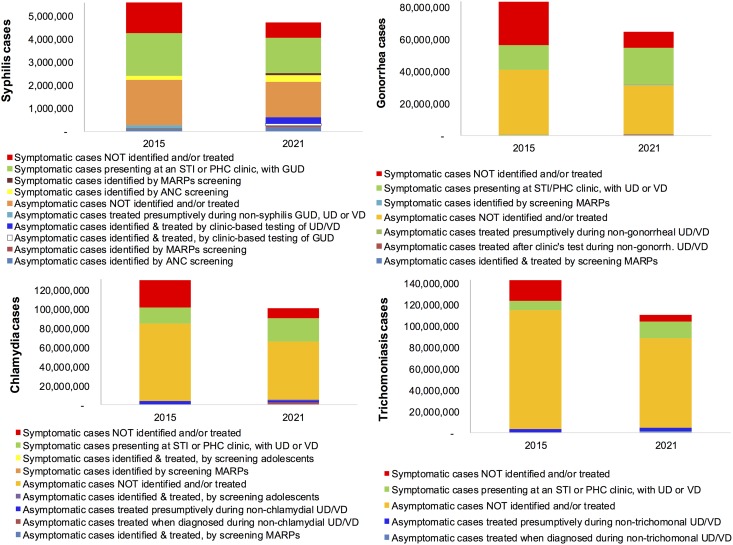
Numbers of curable STI episodes, by diagnostic and treatment status, 117 low- and middle-income countries. Estimated based on the WHO’s 2012STI burden estimates for 2015, with extrapolation to 2021 reflecting the global STI strategy’s burden reduction and diagnostic and treatment coverage targets, as described in Methods and in Table 1.

**Fig 2:**
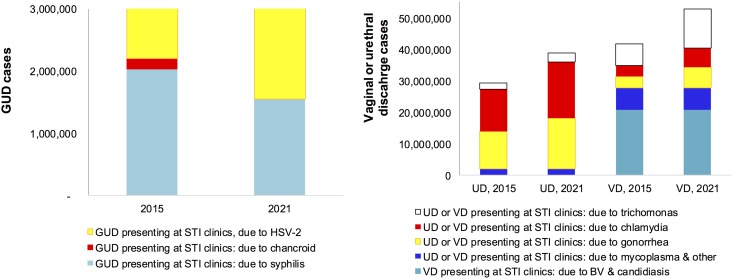
Numbers of genito-urinal syndromes presenting for case management at STI clinics, by diagnostic and treatment status, 117 low- and middle-income countries, 117 low- and middle-income countries. Estimated based on the WHO’s 2012 STI burden estimates for 2015, with extrapolation to 2021 reflecting the global STI strategy’s burden reduction and diagnostic and treatment coverage targets, as described in Methods and in Table 1. The estimated annual GUD cases total 16.2 million in both 2015 and 2021; the y-axis was capped at a lower maximum for readability of the shown sub-sets of cases.

Based on WHO-estimated annual incidences of (symptomatic and asymptomatic) syphilis (Fig 1) and herpetic ulcers, and assuming that these are the predominant etiologies of GUD, we estimated that in 2015 globally there were 8.5 million GUD cases in men and 9.9 million GUD cases in women presenting for clinical treatment (Table 1). If the Strategy achieves a 22% decline in (total) syphilis cases by 2021, then GUD cases will decline to 8.4 million in men (of which 11% due to syphilis) and 9.4 million in women (of which 7% due to syphilis; Fig 2).

Based on WHO-estimated annual incidences of (symptomatic and asymptomatic) gonorrhea, chlamydia and trichomonas, we estimated that global UD cases totaled 29 million in 2015, of which 41% due to gonorrhea, 46% chlamydia, 7% trichomonas and 7% mycoplasma. With the Strategy-based expected declines in gonorrhea, chlamydia and trichomoniasis numbers but increased clinic attendance for symptomatic episodes of these STIs, the number of UD cases presenting for treatment would increase by 2021 to 39 million (Fig 2), of which 42% due to gonorrhea, 46% chlamydia, 7% trichomonas and 5% mycoplasma.

Of an estimated 42 million VD cases in 2015, 9% and 8% would be due to gonorrhea and chlamydia, 17% due to trichomonas and Mycoplasma each, and 50% due to BV. With the Strategy-based expected declines in gonorrhea, chlamydia and trichomoniasis numbers but increased clinic attendance for symptomatic episodes of these STIs, the number of VD cases presenting for treatment would increase by 2021 to 53 million, of which 12% due to gonorrhea, 11% chlamydia, 24% trichomonas, 13% Mycoplasma and 39% BV (Table 1).

### STI service needs

The Strategy’s milestones of 70% global coverage with recommended STI screening, diagnosis and treatment services imply some marked shifts over 2015–2021 in the number of STI cases remaining untreated, versus being treated based on etiologic diagnosis and screening or syndromic management (Fig 1).

For syphilis, screening of ANC clients, FSW and MSM, as well as STI clinic patients attending with non-syphilis conditions, at increasing coverage, increases the overall proportion treated (among symptomatic + asymptomatic cases combined) from 41% in 2015 to 50% in 2021 (Fig 1). The shift from syndromic to etiologic management reduces the number of penicillin treatments administered to non-syphilis GUD patients (compared to 2015 practice), thus saving cost.

For gonorrhea, overall treatment coverage will increase from 20% to 37% due to the scale up of laboratory screening of key population and STI clinic patients attending for non-gonorrheal GUD, alongside scale-up of clinical treatment coverage. Similarly, treatment coverage for chlamydia will increase, from 16% to 30%, due to scaled-up screening among FSW, MSM and STI patients, and (at lower yield) adolescents, as well as scale-up in clinical treatment coverage. Trichomonas treatment coverage will also increase, from 9% to 18%, due to increased treatment coverage and through treatment following etiologic diagnosis in UD and VD patients. By contrast, the clinical burdens and treatment volumes for herpetic ulcers, bacterial vaginosis and mycoplasma infections remain unchanged.

The scale-up of STI screening and treatment for gonorrhoea and chlamydia implies more clinic visits with UD and VD (Fig 1). For syphilis, in contrast, the number of patients presented with GUD to clinics decreases slightly, due mainly to an increasing number of women being identified and treated firstly via ANC screening, which by 2021 would identify 22% of all (symptomatic and asymptomatic) syphilis cases, compared to 11% in 2015 (Fig 1). Overall numbers of patients with GUD (of all etiologies combined) presenting to clinics fall only slightly (from 8.5 million to 8.3 million in men, and from 9.9 million to 9.4 million in women, Table 1), since we assumed no change in herpetic ulcers or their clinical attendance, which make up the vast majority of GUD (88% in 2015, and 91% in 2021).

### Global and regional costs

Over the 6 years, the Strategy global cost is estimated at US$ 18.1 billion (Table 4). Key cost drivers are HPV vaccination (US$ 3.26 billion), HPV screening (US$ 3.69 billion), adolescent chlamydia screening (US$ 2.54 billion), and syphilis screening in ANC (US$ 1.4 billion). Clinical STI management is costed for an overall US$ 3.0 billion, of which service delivery makes up US$ 818 million, and NAAT testing for gonorrhea and chlamydia US$ 1.4 billion.

**Table 4:** Service needs and costs for implementation of the global STI control strategy 2016–2021 across low and middle-income countries.

Action / Intervention:		Unit	Need						Target	Cost per unit & year, US$	Cost all LMIC, 2016–2021 (US$)
			2016	2017	2018	2019	2020	2021			
**Strategic Direction 1: Information for Action**											
**1. Strategic information for advocacy and investment**											
Guidelines and technical support		1 guideline	1							300,000	
**3. National STI surveillance**											
Conduct routine case reporting and periodic prevalence assessment of core STI to assess magnitude of STI problem in target populations including by disaggregating the data.		1 LMIC	120						70%	265,000	**28,560,000**
Conduct routine monitoring of gonococcal antimicrobial resistance and periodic assessment of STI syndrome etiologies		Global lump-sump								1,477,650	**7,586,767**
Strengthen national STI laboratory capacity through quality assurance and introduction of Point-of-Care STI diagnostics to monitor STI and antimicrobial resistance to Neisseria gonorrhea		1 LMIC	120						70%	100,000	**8,400,000**
Guidance and tools		Global lump-sump									
**Strategic Direction 2: Interventions for Impact**											
**2. Reduce vulnerability and risk, especially among key populations**											
Prevention services for adolescents		1 adolescent 15–19 years	678,250,241	691,815,246	705,651,551	719,764,582	734,159,873	748,843,071	70%	0.2	**598,987,839**
Guidelines on sexual violence		1 guideline	1							300,000	
Guidelines on positive gender norms and attitudes to reduce risk		1 guideline	1							300,000	
**3. Prevent STI transmission and acquisition**											
Prevention		1 FSW and/or MSM	26,149,111	26,672,094	27,205,535	27,749,646	28,304,639	28,870,732	70%	0.1	**11,546,62**
Condom programming		Global lump sum									
Advocate / communication		Global lump sum									
**4. Achieve early diagnosis of STIs and linkage to treatment**											
Guidelines		1 guideline	1							300,000	
WHO prequalification		Global lump sum	1							250,000	
**5. Manage symptomatic patients**											
Development of guidelines		1 LMIC	1						70%	5,000	**3,500**
Establish systems to implement management guidelines		1 LMIC	1						70%	5,000	**3,500**
Syphilis/Penicillin treatment for GUD cases at STI clinics			18,408,626	18,408,626	18,408,626	18,178,716	17,948,806	17,718,896	6%	1.7	**11,227,265**
Syphilis testing for GUD cases at STI clinics			18,408,626	18,408,626	18,408,626	18,178,716	17,948,806	17,718,896	70%	0.4	**30,540,243**
Syphilis testing for VD/UD cases at STI clinics			71,525,334	71,525,334	71,525,334	78,317,171	85,109,009	91,900,847	70%	0.4	**131,572,848**
HSV-2/Acyclovir treatment for GUD cases at STI clinics			18,408,626	18,408,626	18,408,626	18,178,716	17,948,806	17,718,896	70%	1.05	**80,168,139**
Gonorrhea/Ceftriaxone treatment for VD/UD cases at STI clinics			71,525,334	71,525,334	71,525,334	78,317,171	85,109,009	91,900,847	56%	0.7	**187,157,748**
Chlamydia/Azithromycin treatment for VD/UD cases at STI clinics			71,525,334	71,525,334	71,525,334	78,317,171	85,109,009	91,900,847	34%	1.0	**153,153,853**
Trichomoniasis/Metronidazole treatment for VD/UD cases at STI clinics			71,525,334	71,525,334	71,525,334	78,317,171	85,109,009	91,900,847	63%	0.05	**14,712,059**
BV/Candidiasis treatment for VD/UD cases at STI clinics			42,035,809	42,035,809	42,035,809	58,657,488	75,279,167	91,900,847	70%	0.3	**70,828,917**
NAAT (= gonorrhea & chlamydia) testing for VD/UD cases at STI clinics			71,525,334	71,525,334	71,525,334	78,317,171	85,109,009	91,900,847	30%	10	**1,409,709,084**
Wet-mode (= trichomoniasis) testing for VD/UD cases at STI clinics			71,525,334	71,525,334	71,525,334	78,317,171	85,109,009	91,900,847	28%	1.0	**129,223,333**
Treatment service delivery for GUD+UD+VD patients			89,933,960	89,933,960	89,933,960	96,495,888	103,057,815	109,619,743	70%	10.0	**629,537,720**
Diagnosis service delivery for GUD+UD+VD patients			89,933,960	89,933,960	89,933,960	96,495,888	103,057,815	109,619,743	70%	3.0	**188,861,316**
Guidelines		1 guideline	1							300,000	
Research in STI case management		Global lump sum	1							500,000	
**6. Reach sex partners and offer them treatment**											
Develop and implement partner management		1 LMIC / project	120						50%	10,000	**600,000**
Guidelines		1 guideline	1							300,000	
**7. Package interventions for maximum impact**											
ANC syphilis	Screening	1 ANC attendee	107,144,889	109,287,787	111,473,543	113,703,014	115,977,074	118,296,616	70%	0.4	**1,383,597,090**
	Treatment of pregnant women found syphilis-infected	1 syphilis-infected pregnant woman	617,077	617,077	617,077	570,797	524,516	478,235	66.5%	11.7	**26,532,625**
Guidelines		1 guideline	1							300,000	
Research new technologies for screening		Global lump sum									
Implementation research: barriers		Global lump sum									
**HPV and Hepatitis B vaccines**											
HPV vaccination programme		10-year old girl, in 102 LMIC	49,000,000	49,735,000	50,232,350	50,734,674	51,242,020	52,000,000		30	**3,255,478,660**
HPV vaccination programme		Target (vaccine & screening)	10%	20%	30%	40%	50%	60%	Vaccination & treatment		
HPV screening & preventive treatment		Woman 30-49y screened, in 102 LMIC	8,100,000	16,524,000	25,281,720	34,383,139	43,838,502	53,658,327	2x / woman lifetime	20.22	**3,692,210,039**
Policies on vaccination		1 policy brief	0	1	1	1	1	1		200,000	
Operational research		1 research project	0	1	1	1	1	1		300,000	
**Control the spread and impact of GC antimicrobial resistance**											
Research on antimicrobial resistance testing		1 research project	1			1				300,000	
**8. Ensuring quality of care for STI services and interventions**											
**Strengthen the continuum of prevention, diagnosis, treatment and care**											
Training		1 Quality-of-Services project, in a LMIC	120						70%	50,000	**4,200,000**
Operational and programmatic guidance		1 guideline	1							500,000	
**Link and integrate services and programmes**											
Linkages and integration models		1 linkage/integration project, in a LMIC	120						10%	20,000	**2,400,000**
Guidelines		1 guideline	1							300,000	
Document and disseminate best practices of integration		Supporting Linkages project	1							500,000	
**Strategic Direction 3: Delivering for human rights, gender equality and health equity**											
**1. Reach key populations with appropriate services**											
Services for Key Populations	Syphilis screening	1 FSW, screened for syphilis	10,488,359	10,698,127	10,912,089	11,130,331	11,352,937	11,579,996	70%	0.12	**5,728,954**
	CT+NG screening	1 FSW, screened for CT & NG	10,488,359	10,698,127	10,912,089	11,130,331	11,352,937	11,579,996	70%	10	**463,132,876**
	Syphilis screening	1 MSM + Trans-Gender, screened for syphilis	15,660,752	15,973,967	16,293,446	16,619,315	16,951,702	17,290,736	70%	0.12	**8,554,219**
	CT+NG screening	1 MSM or Trans-Gender, screened for CT & NG	15,660,752	15,973,967	16,293,446	16,619,315			70%	10	**691,529,424**
	Syphilis treatment, FSW & MSM & Transgender diagnosed by screening		242,187	242,187	242,187	224,023	205,859	187,695	70%	11.7	**10,961,444**
	NG treatment, FSW & MSM & Transgender diagnosed by screening		345,981	345,981	345,981	320,033	294,084	268,136	70%	10.7	**14,399,076**
	CT treatment, FSW & MSM & Transgender diagnosed by screening		800,700	800,700	800,700	740,647	680,595	620,542	70%	11.0	**34,062,373**
Services for adolescents	CT screening	1 adolescent 15–19 years sexually active	804,273,258	820,358,723	836,765,898	853,501,216	870,571,240	887,982,665	5%	10	**2,536,726,500**
	CT treatment, adolescents diagnosed by screening		1,387,371	1,415,119	1,443,421	1,320,685	1,197,949	1,075,213	70%	11.0	**60,091,746**
Guidelines on essential package for key populations and adolescents		1 guideline	1							500,000	
Synthesize and disseminate evidence on prevention and response to violence		1 guideline		1						500,000	
**Strategic Direction 4. Financing for sustainability**											
**1. Develop innovative financing and funding approaches**											
Costing the implementation of the strategy		1 LMIC	120						70%	5,000	**420,000**
Support countries to develop investment cases and funding proposals		Global lump sum									
**Strategic Direction 5. Innovation for acceleration**											
**1. Optimize STI prevention**											
Point-of-Care STI test		Annual lump sum	1	1	1	1	1	1		300,000	
STI vaccines		Annual lump sum	1	1	1	1	1	1		100,000	
Operational research on vaccine introduction		Annual lump sum	1	1	1	1	1	1		100,000	
Development of effective microbicides		Annual lump sum	1	1	1	1	1	1		100,000	
**Global total, STI Activities**											**15,882,405,780**
Add-on Program support cost		14%									2,223,536,809
**Global total, STI Activities + Program support**											**18,105,942,589**

Actions/interventions are listed in the order as in the draft WHO global strategy [1]. Costing excluded STI-related activities that are typically spearheaded by other health programs and for which the WHO has formulated dedicated global health sector strategies (see S1 Appendix).

Global costs increase from US$ 2.6 billion in 2016 to US$ 4.0 billion in 2021, driven by incremental scale-up of HPV vaccination and treatment, and by an increasing population of adolescents 15–19 year-olds of which 5% are to be screened for chlamydia annually.

Sub-Saharan Africa, bearing 40% of global STI burden, covers 44% of the need for STI services (including prevention services, which were costed based on adult population sizes), and 30% of global STI control cost (Table 5). The Western Pacific region, for 15% of global STI burden, makes up 15% of the need for STI services, and 26% of global STI control cost. South-East Asia region covers 20% of global STI burden and 18% of global cost.

**Table 5:** Service needs and costs (millions of US $) for STI service implementation in low- and middle-income countries, by WHO region and national income group.

Region	STI burden share	Share in service needs & in Strategy cost for services at fixed cost (based on share in STI burden)				Share in STI strategy costs—components with varying unit costs (based on share in STI burden & relative cost)				Share in overall STI Strategy cost			
		Low-income	Lower-middle	Upper-middle	All LMIC	Low-income	Lower-middle	Upper-middle	All LMIC	Low-income	Lower-middle	Upper-middle	All LMIC
Africa	40%	24%	16%	4%	**44%**	3%	7%	8%	**18%**	12.3%	11.1%	6.2%	**30%**
America	12%	0.2%	0.8%	6.5%	**8%**	0.8%	4.9%	15.1%	**21%**	0.5%	3.1%	11.2%	**15%**
Eastern Mediterranean	8.9%	0.7%	6.1%	2.5%	**9%**	0.2%	2.4%	3.4%	**6%**	0.4%	4.1%	3.0%	**8%**
Europe	4.3%	0%	0.7%	1.0%	**2%**	0%	1.7%	4.2%	**6%**	0%	1.3%	2.8%	**4%**
South-East Asia	20%	0.8%	21%	0.7%	**22%**	0.6%	8%	5.8%	**14%**	0.7%	13.6%	3.5%	**18%**
Western Pacific	15%	0.2%	2.4%	13%	**15%**	1.4%	8.7%	25%	**35%**	0.9%	5.8%	19.4%	**26%**
**All LMIC**	**100%**	**26%**	**47%**	**27%**	**100%**	**6%**	**32%**	**62%**	**100%**	**15%**	**39%**	**46%**	**100%**
Africa	US$ M:	1,968	1,332	328	**3,629**	270	688	792	**1,749**	2,237	2,021	1,120	**5,378**
America	US$ M:	14.9	67.7	536	**619**	76.5	486	1,495	**2,057**	91.4	554	2,031	**2,677**
Eastern Mediterranean	US$ M:	55.2	500	206	**761**	23.7	241	340	**604**	78.9	740	547	**1,366**
Europe	US$ M:	-	57.1	84.5	**141**	25.9	173	420	**618**	25.9	230	504	**760**
South-East Asia	US$ M:	64.1	1,707	55.0	**1,825**	57.0	762	574	**1,393**	121.1	2,469	629	**3,219**
Western Pacific	US$ M:	16.6	95.2	1,045	**1,257**	140	856	2,476	**3,472**	156.2	1,051	3,522	**4,729**
**All LMIC**	US$ M:	**2,119**	**3,859**	**2,256**	**8,235**	**592**	**3,206**	**6,096**	**9,895**	**2,711**	**7,065**	**8,353**	**18,129**

Across all LMIC, 26% of service delivery volumes/need and 15% of costs are in low-income countries; 47% of volumes/need and 39% of cost in lower-middle income countries, and 27% of volumes/need and 46% of cost in upper-middle income countries (Table 5).

## Discussion

This analysis is the first comprehensive costing of a global STI control strategy, providing a ballpark cost estimate for global STI control implementation over the coming 5 years, and its distribution across world regions and country income group. To achieve this, we presented the first-ever estimation of common STI syndrome volumes, with stratification according to health care seeking and clinic access patterns.

Drivers of global STI control cost—when ignoring prevention costs shared with HIV/AIDS, hepatitis vaccination shared with immunization programs, and health systems cost—are HPV vaccination and screening, chlamydia screening for adolescents, syphilis screening in ANC and clinical STI case management. Considering these cost drivers, opportunities for cost reduction may lie in lowering prices of HPV vaccines, chlamydia tests used for screening adolescents and risk groups, and tests used for syphilis screening in ANC. The costing foresees considerable reductions in prices (compared to 2014–2015 levels) for HPV vaccines (across all income tiers), and for chlamydia diagnostic tests, assumed to be effective from 2016. Costs presented critically depend on whether these envisaged price reductions will actually materialize, but might even be lower than presented if further price reductions could be achieved within the strategy horizon.

Adolescent chlamydia screening incurs considerable cost, but contributes little to overall chlamydia cases detected, compared to screening of MSM, FSW and to screening and presumptive treatment among STI clinic patients. Yield and efficiency would improve if screening is targeted to those adolescents at highest risk—not all sexually active adolescents indiscriminately but rather those with multiple partners, or focused on geographical settings with high background chlamydia rates.

While the Strategy’s costing assumed the syndromic approach as recommended case management strategy throughout 2021, it in envisioned that beyond 2021, affordable rapid tests for an increased number of the STIs will enhanced case management, shifting from syndrome-based to more etiology-based approaches. Notably, ongoing international investments in the development of Point-of-Care tests are expected to generate future savings by lowering STI diagnostic and screening costs.

### Limitations, and steps to refine STI costing

While based on the best available data and expert knowledge, the presented first-ever global STI costing remains inevitably rather imprecise, as it depended on a cascade of uncertain assumptions—probably more uncertain than for recent global costings for other diseases including HIV/AIDS [58], TB [59, 60] and malaria [61] for which there are country-level estimates of the baseline disease and health system burdens, and their ongoing trend over time. Our estimates of the evolving need for STI case management over 2016–2021 were based on regionally aggregate estimates for 2012 for four curable STIs—which, based on globally averaged etiological patterns derived from a few available recent etiological studies (ignoring probable variations in etiology among countries), we extrapolated to include also episodes of BV and Mycoplasma, which contribute the majority of female genito-urinal syndromes presenting for clinical treatment. Furthermore, the existing STI burden estimates are not specific to countries, and lack estimation of recent and ongoing time trends. Also the probabilities that an STI episode becomes symptomatic were assumed to be the same across all countries, using WHO’s latest global estimates [7]. Further uncertainty lies in the relative STI rates and STI burden shares among high-risk groups, and the extent of clustering of STI episodes in the same people (e.g. clinic patients having multiple concurrent STIs), which are also likely to vary by country but for which data did not allow country-stratified assumptions.

As of 2015, WHO does not use an epidemiological model that causally links the effect of scaling-up STI interventions on STI burden. If the global goal of reducing new HIV infections by 90% is achieved by 2030 [44], it is not unlikely that a similar reduction can be achieved for STIs, where STI-specific prevention and treatment interventions are compounded by HIV-targeted behavioural risk reduction and voluntary male medical circumcision. Future epidemiological modelling should confirm if the STI strategy’s intervention milestones (70% coverage of recommended STI prevention and treatment services, by 2020), alongside HIV control, can indeed accomplish the targeted 90% reduction in STI incidence by 2030 − or would rather accomplish a smaller and slower, or larger and faster reduction.

Our estimates of the need and cost for clinical STI case management (some 20% of the global estimated STI control cost) are most affected by these uncertainties and limitations. In comparison, estimated needs and cost for screening and other STI prevention activities—which have defined populations as their target irrespective of the STI burden and yield—are probably less uncertain in terms of the epidemiological basis—but depend more critically on debatable feasible targets and policies (e.g. which age groups to screen for chlamydia or HPV, and how often) and prices of novel diagnostics and drugs. All STI-related costs, however, share the common limitation that they were not validated against current spending data, which (unlike for e.g. HIV/AIDS and tuberculosis, targeted by vertical control programs) are not routinely tracked through any health program or information system, as these costs are integrated in overall health budget, and out-of- pocket expenses by patients are not tracked at all.

Not included in the costing are additional costs borne by international technical agencies, to support and leverage the innovations in service delivery and deliver technical guidance, support, research and advocacy. For their health sector STI strategy, the WHO has costed these activities over 2016–2021 at about US$ 53 million or an additional 0.3% of the presented global implementation cost [1].

Steps to refine global STI control costing may include:

- Regionally stratify assumptions about STI syndromes and etiologies presenting for clinical management, based on comprehensive meta-analysis of country health information system data and research studies.
- For WHO to articulate coverage targets more specifically for each STI intervention or costed action specifically (laboratory diagnosis, syndromic treatment, diagnosis-based treatment, screening, etc.), stratified by STI or syndrome, gender, and between countries or country groups of different STI epidemiological and health systems profile, as emerging through strengthened STI surveillance [62–64] and annual reporting by countries through the Global AIDS Response progress Reporting System [65];
- Refine country-level stratifications in service needs using, in the short term, country STI burden estimates (for prevalence, incidence and DALYs) from the Global Burden of Disease 2013, for the 4 curable STIs as well as for HSV-2 [57];
- And in the longer term, refine country stratification based on forthcoming country-level STI burden and trend estimates, based on statistical synthesis of national surveillance, survey and research data in the new STI estimation tool built into the Spectrum demographic population projection tool [66–70];
- Explore and validate the relation between coverage scale-up and expected burden reductions using dynamic transmission modelling, such as STDSIM [10] which projects STI trends and intervention impacts (including HIV/AIDS) for African countries [71], and inform such impact modelling with local service effectiveness, outcome and impact evaluation data.
- Refine country costings using country-specific service costs for clinical STI case management [72], apply future-year discounting, and conduct national STI service costing studies including but not limited to new services such as HPV vaccination and chlamydia screening.

### STI control financing

STI control implementation (including HPV screening) is expected to be funded from country domestic resources through health systems; and for HPV vaccination through national immunization programs (with donor support for vaccine procurement, which covers around 70% of vaccination costs in countries eligible for support by the Global Alliance for Vaccines & Immunization (GAVI). Of the projected US$ 3.26 billion global cost for HPV vaccination, US$ 0.79 billion (24%) occurs in GAVI-eligible countries. However, GAVI funds only vaccine procurement in those countries (at an expected US$ 0.5 billion), not service delivery.

The costing did not include activities shared with HIV/AIDS programs, like prevention education and STI screening delivered in the context of HIV prevention. In addition to leveraging HIV prevention budgets, STI initiatives will need to leverage funds from maternal, child and adolescent health and immunization programmes. There is a need for a more integrated response enhancing synergies across programmes. Low-income countries will need (continued, and increasing) donor support, whereas upper-middle income countries could be expected to mobilize required funding internally, if national STI strategies are articulated and budgeted. Political commitment, backed by financial commitments of both resource-poor and donor countries, is critical.

In conclusion, the presented first estimate of global needs and costs for STI prevention and care to achieve the global STI strategy’s milestones and targets suggest a need to scale-up STI services and increase investment, in middle-income and especially low-income countries. Countries will need to cost their STI responses, commit domestic funding and leverage funding from HIV, MNCH and other programmes and ensure that services are effective and efficient as possible.

To improve and refine future costing estimates, and to gauge the important expected returns on investment in this global health burden, it is critical to strengthen national STI surveillance and service monitoring and evaluation, and pursue more sophisticated epidemiological (transmission-model-based) estimates of STI burdens.

## Supporting Information

S1 Appendix STI-related activities not costed in this study. (DOCX)

## Abbreviations

ANC: Antenatal care
BV: Bacterial Vaginosis
CT: chlamydia
F: female
FSW: Female Sex Worker
GUD: Genital Ulcer Disease
HPV: Human Papilloma Virus
HSV-2: Herpes Simplex Virus type 2
LMIC: Low- and Middle-Income Countries
M: male
MARPs: most-at-risk-population(s)
MSM: Men having Sex with Men
NAAT: nucleic acid amplification test
NG: gonorrhea
PHC: Primary Health Care
RPR: Rapid Plasma Reagin (syphilis diagnostic test)
STI: Sexually Transmitted Infection(s)
UNAIDS: UD = Urethral Discharge
VD: Vaginal Discharge
WHO: World Health Organization
