# Supplementary material for: Costing of National STI Program Implementation for the Global STI Control Strategy for the Health Sector, 2016-2021
Source: PLoS One. 2017 Jan 27;12(1):e0170773. doi: 10.1371/journal.pone.0170773 (PMC5271339; doi:10.1371/journal.pone.0170773)
Supplement: S1 Appendix — (DOCX) [file pone.0170773.s001.docx]

**S1 Appendix. STI-related activities not costed in this study**

STI-related activities, listed in the global STI strategy but not costed in this study, are those that are typically spearheaded by other health programs and for which the WHO has formulated dedicated global health sector strategies:

| **Non-STI global health sector strategy** | **STI Strategy’s action, not costed** |
| --- | --- |
| HIV/AIDS | - Risk & vulnerability reduction for key populations; - Comprehensive package of STI interventions for prisoners and prison settings; - Condoms; - Prevention guidelines; - Population sizes for KP and integrate biological surveillance with other programs like BSS in the HIV files - Include contact tracing and treatment of partners; - Link STI surveillance with HIV, 2^nd^-generation HIV surveillance and antimicrobial resistance surveillance; - Advocacy |
| Hepatitis / Immunization | - Hepatitis vaccination |
| Health Systems Strengthening & m-health | - Implement health financing systems, financial protection schemes; - Increase savings through improved planning and more efficient procurement and distribution systems; - m-health technology to increase STI services delivery - Community-based service delivery, including effective and more acceptable models (such as m-health) for reaching key populations, with comprehensive services. Research for improved understandings of health-seeking behaviour. |
| Humanitarian, and/or HIV/AIDS | - Incorporate contingency plans for essential STI services into national STI plans to ensure continuity of STI services in settings of humanitarian concern; - Guidance on the delivery of STI services in settings of humanitarian concern |
| Pharmaceutical industry & donors | - Robust regimens to reduce the risk of drug resistance - New, more effective drugs for treating syphilis, *N. gonorrhoeae* and herpes simplex virus - Research on communication and use of new technologies, and systematic community-based support to improve treatment adherence - Increased operational research to identify effective, efficient, safe and acceptable STI diagnostic and treatment approaches |
